# The Dakin-Carrel Antiseptic Solution

**Published:** 1917-05

**Authors:** M. T. Barrett


### THE DAKIN-CARREL ANTISEPTIC SOLUTION

BY M. T. BARRETT, D.D.S.

When the value of Dakin's solution as an efficient general disinfectant was first announced in the general press, my attention was called to the possibility of its employment in dealing with peridental foci of suppuration, but the exact formula for its production was not then available; other matters, moreover, occupied my time to the exclusion of any real effort to acquaint myself with its precise composition. Although, in the interim, Carrel, who had demonstrated its value in his work in France and Switzerland, had published the full directions for its preparation and employment (*Journ. Am. Med. Association*, v, 67, December 9, 1916, p. 1777), I neglected to make any trial of the solution, clinically or otherwise, until in January of the current year, when my attention was again called to it, and I was asked to apply it in his own case of pyorrhea, by Dr. John J. Riley, of Bluefields, Nicaragua.

Dr. Riley had attempted to use both in general surgery and in connection with a number of severe cases of pyorrhea in his practice in Central America, but had likewise failed to obtain and follow the needed directions for its preparation and use. He found his attempts accompanied by so much local irritation that in spite of the readily recognizable disinfection obtained, he felt it necessary to make the trip from his home to the United States in order to make personal observation of its use. After following the work of Dr. W. O. Sherman, of Pittsburgh, for a time, he was able to personally conduct a number of applications in suitable cases in the German Hospital of Philadelphia, under Dr. John B. Deaver of this city, and asked that I employ it in his own case of pyorrhea and such other cases as might permit its use in trial.

I had the solution prepared in accordance with Dr. Riley's instructions, which followed fairly closely the directions published by Carrel, and at first brought it into contact with the contents of pyorrhea pockets directly, under the microscope. Contact of the solution in full and in reduced strengths was in a few moments followed by a cessation of movements of amœbæ, spirochætæ and motile bacteria in the preparations. The amœbæ were found to change in appearance, becoming more and more hyaloid, the clear ectosarc seeming to increase at the expense of the vitally granular endosarc until the latter almost or quite disappeared. This change in the amœbic parasites was quite like that which has been noted in them in analogous observations of contact of solutions of emetin with these parasites (Kolmer and Smith, *Journ. of Infectious Diseases,* v, 18, No. 3, March, 1916, p. 247). Beginning with weak solutions and rapidly increasing the concentration to the full strength of Dakin's solution, as it was found that no unpleasant effects were produced in Dr. Riley's case, the suppuration was soon in abeyance, and shortly ceased entirely as far as gross observation indicated, the attendant redness and swelling of the gums disappearing at the same time, but without actual closure of the pockets in healing.

I immediately made use of the remedy in other cases of pyorrhea with equally satisfactory results, and thereafter also employed it in cases of apical abscesses

—at first in several with fistulous openings, and afterward in apical absceses closed save for the intradental route. In one of the latter, mildly painful symptoms lasting for twenty-four or more hours were complained of by the patient; with this single exception, however, all of the patients were without evidence of local irritation, whether the focus of suppuration was an open or a closed one, and in all the suppuration to gross appearance was eliminated. Dr. E. R. Sausser and Dr. W. J. McKinley of this city have been kind enough to carry on collateral experiments in their practices, and they report to me similar favorable results.

Whether the use of a remedy in which the important agent is essentially free chlorin, with perhaps associated chlorin salts in the form of hypochlorites, chlorites, chorates, and sodium chlorid, will have any as yet unappreciated harmful effects must of necessity be left unanswered for the present. The question of immediate local irritation —when the solution is introduced solely into the pyorrhea pockets and when avoidance of bringing it in contact with the oral mucous membrane is carefully sought—may be set aside, from the statements above made, as apparently in confined foci the chance of immediate irritative effects is trivial, if existing at all. What influence the free chlorin might have on the calcium of the tooth structure is not determined; it is probably at most trivial, if existent. Its action upon gold and amalgam fillings is also *nil*. That it is a powerful antiseptic and disinfectant when it is used as a fresh and proper solution may undoubtedly be accepted. But it is to be remembered that the Dakin solution is not stable, and rapidly loses its efficient free chlorin, even after standing for but a few days, especially if exposed to light and warmth. It should be available in fresh solution; if kept at all, it should be kept in a colored bottle and in a dark and comparatively cool place. It is irritant upon the broken skin and mucous membranes, and it is well to lightly anoint the free surface about a pyorrhea pocket with vaselin before introducing the solution, to protect against the probable overflow of the solution before it becomes diluted by the mouth fluids to a harmless degree.

For a detailed statement of the method of preparation, reference may be made to Dr. Carrel's letter to the *Journal of the American Medical Association* above referred to. Simplified methods of manufacture, avoiding the careful determination Dakin and Carrel advise of the strength in available chlorin of a given specimen of chlorinated lime, and the exact separation of the chlorin of the solution by sodium bicarbonate and neutralization by boric acid, have been proposed—as recently in the *Journal of the American Pharmaceutical Association*, as follows:

| | |
|---|---|
| Sol. chlorinated soda (U.S.P.IX) . . . | 200 cc. |
| Sterile water . . . . . . . . . . . . . . . . . . . . | 800 cc. |
| Boric acid . . . . . . . . . . . . . . . . . . . . . . . | 4 grams. |

Yet it would be well, in trying this or any other remedy in new lines of use, to adhere strictly to the prescribed methods of preparation of the agent, from the standpoints both of safety to the patient and proved efficiency of action, and also that of proper scientific observation and comparison. Any competent pharmaceutical chemist can follow with ease Dr. Carrel's full instructions, and no difficulty should be met in obtaining the product as required.

Dr. Barrett offers this note for publication in the *Dental Cosmos* in the hope that others may be induced to try this agent, to the end that by collective observations a sound evaluation of its properties may the more quickly be arrived at in dental practice.—April *Cosmos*.

